# Surgical Strategies to Reduce Postoperative Recurrence of Crohn's Disease After Ileocolic Resection

**DOI:** 10.3389/fsurg.2021.804137

**Published:** 2021-12-17

**Authors:** Ian S. Reynolds, Katie L. Doogan, Éanna J. Ryan, Daniel Hechtl, Frederik P. Lecot, Shobhit Arya, Sean T. Martin

**Affiliations:** Department of Colorectal Surgery, St. Vincent's University Hospital, Dublin, Ireland

**Keywords:** Crohn's disease, ileocolic resection, anastomotic techniques, mesenteric excision, resection margins

## Abstract

Postoperative recurrence after ileocaecal resection for fibrostenotic terminal ileal Crohn's disease is a significant issue for patients as it can result in symptom recurrence and requirement for further surgery. There are very few modifiable factors, aside from smoking cessation, that can reduce the risk of postoperative recurrence. Until relatively recently, the surgical technique used for resection and anastomosis had little or no impact on postoperative recurrence rates. Novel surgical techniques such as the Kono-S anastomosis and extended mesenteric excision have shown promise as ways to reduce postoperative recurrence rates. This manuscript will review and discuss the evidence regarding a range of surgical techniques and their potential role in reducing disease recurrence. Some of the techniques have been shown to be associated with significant benefits for patients and have already been integrated into the routine clinical practice of some surgeons, while other techniques remain under investigation. Current techniques such as resection of the mesentery close to the intestine and stapled side to side anastomosis are being challenged. It is looking more likely that surgeons will have a major role to play when it comes to reducing recurrence rates for patients undergoing ileocaecal resection for Crohn's disease.

## Introduction

Crohn's Disease (CD) is a transmural inflammatory bowel disease (IBD) that can affect any part of the gastrointestinal tract from the mouth to the anus, it was initially described in detail by Crohn, Ginzburg and Oppenheimer in 1932 (1). While novel medical therapies likely reduce the risk of requiring surgery or delay the need for surgery in patients with CD, it is still estimated that up to 80% of patients with CD will require surgery during their lifetime (2, 3). Ileocaecal resection with an ileocolic anastomosis is the most frequently performed operation for patients with CD (4). Endoscopic evidence of postoperative recurrence (POR) can be found in 70–90% of patients at 1 year and is typically defined as a Rutgeerts score of i2 or greater (5, 6). Clinical POR is more loosely defined and includes an increase in patients symptoms attributable to recurrent disease including diarrhoea, weight loss and abdominal pain (7). Quality of life questionnaires such as the Inflammatory Bowel Disease Questionnaire and scoring systems such as the Crohn's Disease Activity Score (CDAI) are used as standardised ways to report clinical recurrence (8, 9). Surgical POR is defined by the need for repeat resection (10). The number of patients with clinically significant POR is lower than the number of patients with endoscopic POR. However, ~40% of patients at 5 years and 55% of patients at 10 years require reoperation (11). The presence of perianal CD, extensive intestinal disease and resection of segments of intestine >50 cm are associated with an increased risk of POR (2, 12). Continuing to smoke after surgery is associated with a higher risk of relapse whereas former smokers and those who quit smoking have similar relapse rates to non-smokers (13). The use of postoperative anti-TNF agents has been shown to reduce the risk of endoscopic POR and a randomised controlled trial from Yoshida et al. demonstrated a lower risk of clinical POR in those treated with infliximab compared to a control group (14–17).

While patient modifiable factors and CD medications have been studied extensively as strategies to decrease POR there is a growing body of evidence regarding potential intraoperative surgical techniques that might play a role in reducing POR after ileocolic resection for CD. The role of surgical resection margins, anastomotic techniques and configurations as well as extended mesenteric excision have all been evaluated as potential mechanisms to reduce endoscopic, clinical and surgical POR in the setting of CD (18). This review will describe and summarise the evidence surrounding each of these techniques with a particular focus on anastomotic techniques and configurations.

## Resection Margins

The role of resection margins in Crohn's disease was extensively studied in the 1980s and 1990s with numerous studies reporting a range of conflicting results (19–29). In order to get definitive answers to this question one must refer to the randomised controlled trial published by Fazio et al. in 1996 (30). In this study 75 patients were randomised to a limited resection (proximal line of resection 2 cm from the limit of macroscopically diseased bowel) and 56 patients were randomised to an extended resection (proximal line of resection 12 cm from the limit of macroscopically diseased bowel). The surgical recurrence rate was 25.3% in the limited resection group compared to 17.9% in the extended resection group (*p* = 0.31). Similarly, no significant differences were found in clinical recurrence rates between the groups (*p* = 0.56). While this study closed the issue regarding macroscopic resection margins with most in agreement that resecting wide margins of macroscopically normal bowel was not necessary, the issue of microscopic resection margins remained unanswered (31). Determining whether or not the margin is histologically free from disease is not always straight forward as it may have features of non-specific inflammation as opposed to features of CD. There are a number of retrospective studies that have looked at the association between histologically disease free margins and recurrence and yet again the results are conflicting. The original paper by Pennington et al. from Johns Hopkins that sparked the debate was published in 1980 and found no difference in recurrence between those with histologically involved and uninvolved margins (26). The findings from this paper were soon challenged by a retrospective study published in 1983 by Wolff et al. from the Mayo Clinic that showed an increased rate of recurrence in those with histologically involved margins (28). A larger retrospective study published in 1991 by Kotanagi et al. from the Cleveland Clinic found no association between histologically involved margins and recurrence (32). A prospective randomised controlled trial by McLeod et al. found no difference in recurrence rates between those with and those without histologically involved margins (33). While the evidence is equivocal, it appears that overall the majority of publications favour the theory that histologically involved margins do not increase the risk of recurrence after ileocolic resection for CD (34–36). It is worth noting that the topic has continued to be investigated and some recent studies, including a meta-analysis, have again suggested that an association exists between histologically positive margins and recurrence (37, 38). There still remains issues with how histological margin positivity and recurrence are reported. There is currently not enough evidence to suggest that intraoperative frozen sections should be assessed by pathologists and wider resections be undertaken if the margins are histologically positive. The significance of margin positivity remains undetermined but it may play a role when deciding whether or not to commence a patient on postoperative prophylactic therapy.

## Laparoscopic vs. Open Ileocolic Resection

The role of laparoscopic surgery in CD has been studied extensively and the initial studies set out to examine the feasibility of laparoscopic resection for ileocolic Crohn's (39–42). Once laparoscopic surgery was deemed to be safe and feasible in the setting of CD, further studies went on to examine the potential benefits to patients and they identified that patients who underwent laparoscopic surgery had reduced length of ileus, shorter duration to commencing diet, reduced narcotic use, faster recovery of pulmonary function, reduced rates of complications, shorter length of stay, lower 5-year small bowel obstruction rates, fewer incisional hernias and reduced overall costs associated with their care (43–47). More recently studies have focused on the recurrence rates of CD after laparoscopic resection compared to open resection. From the data published to date, it appears that there is no significant difference in endoscopic, clinical or surgical recurrence rates between patients who undergo open or laparoscopic resection for ileocolic CD (48–54). Despite the fact that there is no benefit in relation to disease recurrence with laparoscopic surgery for ileocolic CD, this technique is still the recommended first line approach, where available, by the European Crohn's and Colitis Organisation (ECCO) given the other benefits, highlighted above, that it offers to patients. In the absence of available laparoscopic expertise emergency operations should not be delayed (55).

## Anastomotic Techniques and Configurations

### Side to Side Anastomosis vs. End to End Anastomosis

The bulk of the literature to date regarding anastomotic techniques pertains to studies of side to side anastomosis (SSA) compared to end to end anastomosis (EEA). The literature regarding these two techniques needs to be interpreted with caution as many of the studies are retrospective in nature and include a mix of both stapled and handsewn anastomoses (11, 56–62). A meta-analysis from He et al. published in 2014, that included 821 patients from eight studies compared handsewn end to end anastomosis (HEEA) (*n* = 424) and stapled side to side anastomosis (SSSA) (*n* = 396) (63). They found that the rate of overall short-term postoperative complications, the recurrence rate and the reoperation rate were all lower in the SSSA group. No difference was identified in the length of hospital stay or mortality between the groups. A systematic review and meta-analysis from Feng et al. published in 2018, that included 1,113 patients from 11 studies, also compared HEEA and SSSA (64). The authors demonstrated that SSSA was associated with an overall reduction in postoperative complications, clinical recurrence and the need for reoperation. No difference was identified in postoperative length of stay or mortality between the two techniques. Another meta-analysis by Guo et al. published in 2013 that compared handsewn and stapled SSA to other anastomotic techniques (handsewn EEA, handsewn end to side, stapled EEA & handsewn end to side) found a lower overall rate of postoperative complications in the SSA group and no difference in endoscopic recurrence, symptomatic recurrence and reoperation rates between the groups (65). As the above meta-analyses all include retrospective studies it is important to evaluate the available randomised controlled data on an individual level. A study by McLeod et al. published in 2009 randomised 170 patients to either SSSA or HEEA and 139 patients were eventually included in the efficacy analysis (66). The mean duration of surgery was shorter in the SSSA cohort (113 vs. 138 min, *p* = 0.0009), however, there were no differences in postoperative length of stay (6 days vs. 6 days), complication rates (24 vs. 20%, *p* = 0.79), leak rates (7 vs. 7%, *p* = 0.86) and re-operative rates (7 vs. 7%, *p* = 0.86). The endoscopic (37.9 vs. 42.5%, *p* = 0.55) and clinical (22.7 vs. 21.9%, *p* = 0.92) recurrence rates were similar between the SSSA and HEEA cohorts respectively. Another randomised controlled trial published in 2013 by Zurbuchen et al. was terminated early due to insufficient patient recruitment (67). The authors randomised 36 patient to SSSA and 31 patients to HEEA. They concluded that there was no difference in early postoperative outcomes between the two types of anastomoses and they did not have sufficient data to make a statement regarding a difference in perianastomotic recurrence rates between the two techniques. There is probably insufficient data available that allows one to draw firm conclusions as to which type of anastomosis is superior, SSSA or HEEA (68). There does however appear to be a definite trend towards worse short term outcomes for HEEA compared to other types of anastomoses, however a single study has demonstrated better quality of life and reduced hospitalisation at 2 years postoperatively in patients who underwent HEEA compared to those who underwent SSSA (69, 70). As the overall evidence appears to be in favour of SSSA over HEEA, this is currently the technique recommended by the ECCO (55). The question as to whether a stapled SSA is superior to a sutured SSA remains to be answered (71–73).

### Kono-S Anastomosis

The Kono-S anastomosis was first described by Kono and colleagues in 2011 and is the anastomotic technique that has attracted the most attention in recent years (74). With this technique the mesentery of the affected intestinal segment is divided at the mesenteric edge of the bowel to avoid devascularisation and denervation of residual bowel, thus preserving blood supply and neural control. The intestine is transected proximal and distal to the diseased segment with a linear stapler ensuring that the staple line is equidistant from the mesenteric and anti-mesenteric borders and perpendicular to them. Both ends of the remaining stapled intestine are sutured together with 4 or 5 sutures to create a supporting column. Longitudinal enterotomies are made on the antimesenteric aspect of the intestine 1 cm from the supporting column. The enterotomies should be big enough to allow a transverse lumen size of 7–8 cm. The anastomosis is then created in a transverse fashion using a running 3/0 resorbable suture (75, 76). The initial retrospective study from Kono et al. compared 69 patients who underwent the Kono-S anastomosis between 2003 and 2009 to 73 patients who underwent a HEEA or a stapled or sutured SSA between 1993 and 2003. They found that the median endoscopic recurrence score in the Kono-S group was less than in the conventional anastomosis group, the risk of reoperation was also lower in the Kono-S group (0 vs. 15%, *p* = 0.0013) (74). Kono et al. then reported similar findings in a group of 187 patients who underwent Kono-S anastomosis in both Japan and the USA (77). The 5 and 10-year cumulative surgical recurrence rate were both 1.7%, with surgical recurrence occurring in only two Japanese patients. Since then a number of case reports, case series and retrospective studies with comparison to historical patients undergoing traditional anastomotic techniques have been published and demonstrated encouraging results (78–82). A retrospective study by Shimada et al. published in 2019 compared 117 patients who underwent Kono-S anastomosis to 98 patients who underwent HEEA (83). Patients in the Kono-S group had a lower rate of surgical recurrence (3.4 vs. 24.4%) and the 5-year surgery free survival rate at the anastomosis site was better with the Kono-S anastomosis compared to HEEA (95.0 vs. 81.3%, *p* < 0.001). Luglio et al. published a randomised controlled trial in 2020, The SuPREMe-CD Study, comparing the Kono-S anastomosis to SSSA (84). This study randomised 36 patients to the Kono-S anastomosis and 43 patients to SSSA and had a primary endpoint of endoscopic recurrence at 6 months and secondary endpoints of clinical recurrence at 12 and 24 months, endoscopic recurrence at 18 months and surgical recurrence at 24 months. The endoscopic recurrence rate at 6 months was lower in the Kono-S group compared to the SSSA group (22.2 vs. 62.8%, *p* < 0.001). Clinical recurrence was similar between the two groups at 12 months (8 vs. 18%, *p* = 0.2), however, it was lower in the Kono-S group at 24 months (18 vs. 30.2%, *p* = 0.04). The time until clinical recurrence was longer in the Kono-S group (*p* = 0.037). Surgical recurrence rates at 24 months were similar between the two groups (0 vs. 4.6%, *p* = 0.3). In summary this trial demonstrated that the Kono-S anastomosis was associated with lower rates of endoscopic and clinical recurrence with a similar safety profile to SSSA. A number of systematic reviews and meta-analyses have been published and appear to confirm the lower anastomotic leak rates and lower rates of endoscopic and surgical recurrence reported in the individual studies (85–87). There is an ongoing prospective multicentre randomised controlled trial (NCT03256240) comparing the Kono-S anastomosis to SSSA, this will hopefully give more definitive answers on the impact of the Kono-S anastomosis on the natural history of CD after ileocolic resection.

### Ileocolic Nipple Valve Anastomosis

Several other anastomotic techniques have been described in the literature that have attempted to reduce recurrence rates in patients undergoing ileocolic resection for CD. One such technique is the ileocolic nipple valve anastomosis (INVA). The nipple valve is constructed by everting the distal ileum for a length of 3–4 cm. The eversion is secured with a single layer of interrupted sutures. The anastomosis is then completed by telescoping the nipple into the open colon and joining the colon to the ileum with two rows of interrupted sutures (88). This type of anastomosis is believed to work by preventing reflux of colonic contents back into the neoterminal ileum. *Clostridium perfringens* which is present in colonic contents can facilitate the release of arachidonic acid from enterocytes in the distal ileum, the ileal mucosa also contains phospholipase A_2_ which may be activated by phospholipase C that is contained in refluxed colonic contents. Activated phospholipase A_2_ then catalyses the release of arachidonic acid from various phospholipids which goes on to produce various inflammatory mediators (89–91). The initial paper compared six patients who underwent INVA to 21 patients who underwent HEEA. In the INVA group three out of six patients had symptomatic recurrence at an average of 37 months compared to 16 out of 21 patients at an average of 17 months in the HEEA group. One patient required repeat surgery in the INVA group after an average of 56 months compared to five of 21 patients in the HEEA group after an average of 23 months. Another study followed up 59 patients who underwent INVA and described the clinical and surgical recurrence rates over time, this was a longer follow up study that followed on from a pilot study published previously by the same author (92). The clinical recurrence rate in this study was 24% at 5 years from surgery and the reoperation rate was 16% at 5 years from surgery. The authors concluded that a randomised controlled trial was needed to assess this anastomotic technique further, although it appears from the literature that a trial of this nature was not carried out (93).

## Extended Mesenteric Excision

As our understanding of the anatomy and physiology of the mesentery becomes clearer, the role of the mesentery in health and disease is beginning to unfold (94, 95). In particular the part played by the mesentery in the setting of Crohn's disease is becoming more apparent (96–100). With evidence to suggest that patients who undergo proctectomy for CD with close rectal dissection have more perineal complications (59.5 vs. 17.6%, *p* = 0.007) and lower healing rates (51.4 vs. 88.2%, *p* = 0.014) compared to patients who undergo proctectomy for CD with total mesorectal excision, it seems plausible that extended excision of the mesentery for patients with ileocolic CD might also improve outcomes (101). Interestingly, in the study on proctectomy in CD, eight patients with perineal complications after proctectomy with close rectal dissection underwent repeat surgery in the form of mesorectal excision with omentoplasty. This more radical procedure resulted in complete perineal wound closure in six of the eight patients, perhaps more interesting though was the findings of pro-inflammatory characteristics in the excised mesorectum in these patients even after the rectum had been excised, this surely points towards a role of the mesentery in the pathophysiology of CD. A paper published by Coffey et al. in 2018 compared 34 patients undergoing ileocolic resection with inclusion of the mesentery after August 2010 to 30 patients undergoing ileocolic resection where the mesentery was divided flush with the region of intestine to be resected between January 2004 and April 2010 (102). The cumulative reoperation rate was 2.9% in those undergoing resection with inclusion of the mesentery compared to 40% in those undergoing resection in the group where the mesentery was divided flush with intestine (*p* = 0.003). On multivariable analysis it was found that not resecting the mesentery was an independent predictor of recurrence requiring surgical intervention (*p* = 0.007). No difference was identified between the groups with regard to the length of intestine resected (*p* = 0.198). It is worth noting that the length of follow up was shorter in the group undergoing resection with inclusion of the mesentery and this may have resulted in an underestimation of the number of recurrences (103). A further study by Zhu et al. published in 2021 compared 66 patients with Crohn's colitis who underwent extensive mesenteric excision (EME) to 60 patients who underwent limited mesenteric excision (LME) (104). They found that LME was an independent predictor of postoperative surgical recurrence (HR 2.67, 95% CI 1.04–6.85, *p* = 0.4) and that those in the EME group had a longer postoperative surgical recurrence-free survival time when compared with those in the LME group (*p* = 0.01). Interestingly and unexpectedly, intraoperative blood loss was higher in the LME group (*p* = 0.002). A number of other randomised controlled trials assessing the role of extended mesenteric excision in CD are ongoing (NCT03172143 and NCT03769922).

## Discussion

Clinical recurrence of CD after ileocolic resection is a major issue for patients as it results in recurrence of symptoms, a poorer quality of life and potentially the need for reoperation. There has been a large volume of research focused on disease related factors and patient related factors that might influence the risk of POR. Smoking appears to be the most modifiable patient factor that can influence POR (105). There is mixed evidence regarding the effect of anti-TNF medications on disease recurrence and at present these medications are used selectively after multidisciplinary team discussion in patients deemed to be at a high risk of POR (15, 17). Early efforts at reducing POR using surgical techniques was predominantly focused on extended macroscopic resection margins and the presence of histological disease on the cut margin. Ultimately performing resections with extended margins was deemed to be unhelpful in reducing POR and the current recommendation is that resection with a small amount of normal macroscopic bowel results in the same rate of POR as an extended resection. Similarly, the presence of microscopic disease on the cut margin does not appear to influence POR rates, although there is ongoing research in this area. More recently the focus has shifted to the type of anastomotic technique that is used for ileocolic resections. In summary, SSSA may have some benefits, such as a reduced rate of anastomotic leak, compared to HEEA. However, neither of these techniques seems to confer any advantage over the other when it comes to POR and the rates of endoscopic recurrence when either of these techniques are used is remarkably high (70–90% at 1 year) (5, 6). A novel anastomotic technique, the Kono-S anastomosis, is the first surgical technique that has demonstrated reduced rates of both endoscopic and clinical recurrence. These results have been replicated in a randomised controlled trial (84). Further studies are ongoing to confirm the data that has already been published, if the results are replicated it would mean that the greatest opportunity to reduce POR after ileocolic resection will be in the hands of surgeons. A second exciting technique that might be a valuable tool in reducing POR is extended mesenteric excision. Early data has shown a reduced risk of POR in patients undergoing extended mesenteric excision and there are ongoing randomised controlled trials studying the role of this technique (102, 104). The most exciting ongoing randomised controlled trial is the MEsenteric Excision and Kono-S Anastomosis Trial (MEERKAT) (NIHR131988) which is due to finish in May 2026. This trial is randomising patients to one of four groups; (1) Kono-S anastomosis and radical mesenteric resection; (2) Kono-S and close mesenteric resection; (3) Standard anastomosis and radical mesenteric resection; (4) Standard anastomosis and close mesenteric resection. The results are eagerly awaited as they will likely have a strong influence on future clinical practice.

It will be a significant breakthrough in the management of CD if the Kono-S anastomosis or extended mesenteric resection are definitely proven to be effective at reducing POR after ileocolic resection, particularly given the lack of certainty regarding the efficacy of medications at reducing POR. The evolution of new techniques are coming at an important time when we are likely to see more patients with isolated ileocolic disease being referred for consideration for surgical resection (106–109). The reason for this is based on a number of publications including the LIR!C trial which has demonstrated that laparoscopic ileocaecal resection is cost effective and results in similar quality of life as induction and maintenance treatment with infliximab in patients with non-stricturing limited (affected segment ≤40 cm) ileocaecal CD for whom conventional treatment was unsuccessful (110, 111). The ECCO guidelines also recommend offering laparoscopic ileocaecal resection to patients with limited non-stricturing ileocaecal CD as a reasonable alternative to infliximab (55). The Kono-S anastomosis or extended mesenteric excision might offer the most substantial reduction in the risk of recurrence to date for patients undergoing ileocaecal resection. The relevance of these techniques on our understanding of the pathology of CD is not to be underestimated. While questions have been raised about how a technique that preserves the mesentery seems to have similar benefits to a technique that removes the mesentery, one must remember that these techniques share a common feature in that the diseased mesentery is excluded from the anastomosis. With the Kono-S technique the anastomosis is performed on the antimesenteric side of the intestine and the mesentery is not in contact with the anastomosis at all. With the extended mesenteric excision technique the diseased mesentery is completely removed. These techniques may serve to disrupt mesenchymal and immunological inputs into the intestine which may reduce the number of fibroblast precursors, known as fibrocytes, that can reach the intestine (112). Both of these novel techniques point towards a strong role of the mesentery in CD and in the setting of recurrence after ileocolic resection, further work needs to be done in this area (100, 113).

Finally, this study raises questions about who should be performing CD surgery and where it should take place. It is beginning to look as if the days of a resection with division of the mesentery close to the intestine and a SSSA are numbered. More complex techniques such as the Kono-S anastomosis and extended mesenteric excision appear to be strong contenders to become the recommended techniques for CD surgery going forward. These techniques are technically demanding and potentially dangerous in the hands of surgeons not performing them regularly, particularly given the difficulty associated with handling the Crohn's mesentery. It is likely that most CD resections will take place in centres with a dedicated multidisciplinary IBD team and experienced surgeons trained in a range of evidence based techniques that will allow the patient to achieve the best outcomes possible. A multidisciplinary approach will also ensure adequate follow up for patients which may facilitate early diagnosis of endoscopic recurrence and possibly earlier commencement of medications that may reduce the patients risk of going on to develop a surgical recurrence. Recurrence is common after ileocaecal resection but it should also be considered after any resection in the setting of CD. For those with Crohn's colitis undergoing proctocolectomy the median surgical recurrence rate at 5-years is 16.9%, although interestingly, it is only 5.7% for those without prior small bowel CD (114). While detection of recurrence is typically performed by endoscopic assessment, there is emerging evidence that recurrent small bowel disease in those with stomas may be detected by checking faecal calprotectin levels from ileostomy effluent. A recent study has shown that faecal calprotectin levels of >60 mcg/g from ileostomy effluent has a sensitivity of 87.5%, a specificity of 91.4%, a positive predictive value of 82.3%, a negative predictive value of 94.1% and test diagnostic accuracy of 90.1% (115). Using faecal calprotectin in this manner might be a good way to spare people at low risk of recurrence from repeated invasive testing going forward.

## Conclusions

Postoperative recurrence of Crohn's disease after ileocaecal resection is a frequently encountered problem. It causes significant difficulty for patients and may require repeated resections over time. Aside from ceasing smoking, there are limited modifiable factors available to reduce recurrence after surgery. Advancements in surgical techniques, in particular with the Kono-S anastomosis and extended mesenteric resection are beginning to be supported by promising evidence. While trials are ongoing, these novel techniques might represent the biggest breakthrough in recurrence reducing mechanisms to date. Surgeons with a subspecialist interest in the management of Crohn's disease will likely have new techniques in their armamentarium in the near future.

## Author Contributions

IR: data collection. All authors: study concept and design, study materials, manuscript preparation, and manuscript review.

## Conflict of Interest

The authors declare that the research was conducted in the absence of any commercial or financial relationships that could be construed as a potential conflict of interest.

## Publisher's Note

All claims expressed in this article are solely those of the authors and do not necessarily represent those of their affiliated organizations, or those of the publisher, the editors and the reviewers. Any product that may be evaluated in this article, or claim that may be made by its manufacturer, is not guaranteed or endorsed by the publisher.

## References

[1] Crohn BB Ginzburg L Oppenheimer GD. Regional ileitis: a pathologic and clinical entity. J Am Med Assoc. (1932) 99:1323–9. 10.1001/jama.1932.0274068001900510828911

[2] Bernell O Lapidus A Hellers G. Risk factors for surgery and postoperative recurrence in Crohn's disease. Ann Surg. (2000) 231:38–45. 10.1097/00000658-200001000-00006 10636100 PMC1420963

[3] Lewis RT Maron DJ. Efficacy and complications of surgery for Crohn's disease. Gastroenterol Hepatol (N Y). (2010) 6:587–96. 21088749 PMC2976865

[4] Yamamoto T Keighley MR. Stapled functional end-to-end anastomosis in Crohn's disease. Surg Today. (1999) 29:679–81. 10.1007/BF02483001 10452253

[5] Olaison G Smedh K Sjödahl R. Natural course of Crohn's disease after ileocolic resection: endoscopically visualised ileal ulcers preceding symptoms. Gut. (1992) 33:331–5. 10.1136/gut.33.3.331 1568651 PMC1373822

[6] Rutgeerts P Geboes K Vantrappen G Beyls J Kerremans R Hiele M. Predictability of the postoperative course of Crohn's disease. Gastroenterology. (1990) 99:956–63. 10.1016/0016-5085(90)90613-6 2394349

[7] Connelly TM Messaris E. Predictors of recurrence of Crohn's disease after ileocolectomy: a review. World J Gastroenterol. (2014) 20:14393–406. 10.3748/wjg.v20.i39.14393 25339826 PMC4202368

[8] Irvine EJ. Quality of life of patients with ulcerative colitis: past, present, and future. Inflamm Bowel Dis. (2008) 14:554–65. 10.1002/ibd.20301 17973299

[9] Best WR Becktel JM Singleton JW Kern F Jr.. Development of a Crohnop disease activity index National Cooperative Crohnra Disease Study. Gastroenterology. (1976) 70:439–44. 10.1016/S0016-5085(76)80163-1 1248701

[10] Cunningham MF Docherty NG Coffey JC Burke JP O'Connell PR. Postsurgical recurrence of ileal Crohn's disease: an update on risk factors and intervention points to a central role for impaired host-microflora homeostasis. World J Surg. (2010) 34:1615–26. 10.1007/s00268-010-0504-6 20195604

[11] Muñoz-Juárez M Yamamoto T Wolff BG Keighley MR. Wide-lumen stapled anastomosis vs. conventional end-to-end anastomosis in the treatment of Crohn's disease. Dis Colon Rectum. (2001) 44:20–5. 10.1007/BF02234814 11805559

[12] Bernell O Lapidus A Hellers G. Risk factors for surgery and recurrence in 907 patients with primary ileocaecal Crohn's disease. Br J Surg. (2000) 87:1697–701. 10.1046/j.1365-2168.2000.01589.x 11122187

[13] Nunes T Etchevers MJ García-Sánchez V Ginard D Martí E. Barreiro-de Acosta M. Impact of smoking cessation on the clinical course of Crohn's disease under current therapeutic algorithms: a multicenter prospective study. Am J Gastroenterol. (2016) 111:411–9. 10.1038/ajg.2015.401 26856753

[14] Auzolle C Nancey S Tran-Minh ML Buisson A Pariente B Stefanescu C. Male gender, active smoking and previous intestinal resection are risk factors for post-operative endoscopic recurrence in Crohn's disease: results from a prospective cohort study. Aliment Pharmacol Ther. (2018) 48:924–32. 10.1111/apt.14944 30126030

[15] Regueiro M Feagan BG Zou B Johanns J Blank MA Chevrier M. Infliximab reduces endoscopic, but not clinical, recurrence of Crohn's disease after ileocolonic resection. Gastroenterology. (2016) 150:1568–78. 10.1053/j.gastro.2016.02.072 26946343

[16] De Cruz P Kamm MA Hamilton AL Ritchie KJ Krejany EO Gorelik A. Efficacy of thiopurines and adalimumab in preventing Crohn's disease recurrence in high-risk patients–a POCER study analysis. Aliment Pharmacol Ther. (2015) 42:867–79. 10.1111/apt.13353 26314275

[17] Yoshida K Fukunaga K Ikeuchi H Kamikozuru K Hida N Ohda Y. Scheduled infliximab monotherapy to prevent recurrence of Crohn's disease following ileocolic or ileal resection: a 3-year prospective randomized open trial. Inflamm Bowel Dis. (2012) 18:1617–23. 10.1002/ibd.21928 22081474

[18] Click B Merchea A Colibaseanu DT Regueiro M Farraye FA Stocchi L. Ileocolic resection for crohn disease: the influence of different surgical techniques on perioperative outcomes, recurrence rates, and endoscopic surveillance. Inflamm Bowel Dis. (2021). 10.1093/ibd/izab081. [Epub ahead of print]. 33988234

[19] Wolff BG. Resection margins in Crohn's disease. Br J Surg. (2001) 88:771–2. 10.1046/j.0007-1323.2001.01776.x 11412245

[20] Krause U Ejerblad S Bergman L. Crohn's disease. A long-term study of the clinical course in 186 patients. Scand J Gastroenterol. (1985) 20:516–24. 10.3109/00365528509089690 4023619

[21] Softley A Myren J Clamp SE Bouchier IA Watkinson G de Dombal FT. Factors affecting recurrence after surgery for Crohn's disease. Scand J Gastroenterol Suppl. (1988) 144:31–4. 3165554

[22] Raab Y Bergström R Ejerblad S Graf W Påhlman L. Factors influencing recurrence in Crohn's disease. An analysis of a consecutive series of 353 patients treated with primary surgery. Dis Colon Rectum. (1996) 39:918–25. 10.1007/BF02053992 8756849

[23] Bergman L Krause U. Crohn's disease. A long-term study of the clinical course in 186 patients. Scand J Gastroenterol. (1977) 12:937–44. 10.3109/00365527709181353 605353

[24] Ihász M Mester E Réfi M. Experience in the surgical management of Crohn's disease. Am J Proctol. (1975) 26:47–62. 1163646

[25] Kåresen R Serch-Hanssen A Thoresen BO Hertzberg J. Crohn's disease: long-term results of surgical treatment. Scand J Gastroenterol. (1981) 16:57–64. 7233081

[26] Pennington L Hamilton SR Bayless TM Cameron JL. Surgical management of Crohn's disease. Influence of disease at margin of resection. Ann Surg. (1980) 192:311–8. 10.1097/00000658-198009000-00006 6998388 PMC1344907

[27] Lindhagen T Ekelund G Leandoer L Hildell J Lindström C Wenckert A. Recurrence rate after surgical treatment of Crohn's disease. Scand J Gastroenterol. (1983) 18:1037–44. 10.3109/00365528309181837 6673073

[28] Wolff BG Beart RW Jr. Frydenberg HB Weiland LH Agrez MV Ilstrup DM. The importance of disease-free margins in resections for Crohnon disease. Dis Colon Rectum. (1983) 26:239–43. 10.1007/BF02562486 6839893

[29] Martin G Heyen F Dubé S. [Factors of recurrence in Crohn disease]. Ann Chir. (1994) 48:685–90. 7872615

[30] Fazio VW Marchetti F Church M Goldblum JR Lavery C Hull TL. Effect of resection margins on the recurrence of Crohn's disease in the small bowel. A randomized controlled trial. Ann Surg. (1996) 224:563–71. 10.1097/00000658-199610000-00014 8857860 PMC1235424

[31] Fazio VW Marchetti F. Recurrent Crohn's disease and resection margins: bigger is not better. Adv Surg. (1999) 32:135–68. 9891742

[32] Kotanagi H Kramer K Fazio VW Petras RE. Do microscopic abnormalities at resection margins correlate with increased anastomotic recurrence in Crohn's disease? Retrospective analysis of 100 cases. Dis Colon Rectum. (1991) 34:909–16. 10.1007/BF02049707 1914726

[33] McLeod RS Wolff BG Steinhart AH Carryer PW O'Rourke K Andrews DF. Prophylactic mesalamine treatment decreases postoperative recurrence of Crohn's disease. Gastroenterology. (1995) 109:404–13. 10.1016/0016-5085(95)90327-5 7615189

[34] Adloff M Arnaud JP Ollier JC. Does the histologic appearance at the margin of resection affect the postoperative recurrence rate in Crohn's disease? Am Surg. (1987) 53:543–6. 3674595

[35] McLeod RS. Resection margins and recurrent Crohn's disease. Hepatogastroenterology. (1990) 37:63–6. 2179092

[36] Yamamoto T. Factors affecting recurrence after surgery for Crohn's disease. World J Gastroenterol. (2005) 11:3971–9. 10.3748/wjg.v11.i26.3971 15996018 PMC4502089

[37] Ryan JM Rogers AC O'Toole A Burke JP. Meta-analysis of histological margin positivity in the prediction of recurrence after Crohn's resection. Dis Colon Rectum. (2019) 62:882–92. 10.1097/DCR.0000000000001407 31188190

[38] Poredska K Kunovsky L Marek F Kala Z Prochazka V Dolina J. The influence of microscopic inflammation at resection margins on early postoperative endoscopic recurrence after ileocaecal resection for Crohn's disease. J Crohns Colitis. (2020) 14:361–8. 10.1093/ecco-jcc/jjz153 31501878

[39] Reissman P Salky BA Edye M Wexner SD. Laparoscopic surgery in Crohn's disease. Indications and results. Surg Endosc. (1996) 10:1201–3. 10.1007/s004649900279 8939843

[40] Bauer JJ Harris MT Grumbach NM Gorfine SR. Laparoscopic-assisted intestinal resection for Crohn's disease. Which patients are good candidates? J Clin Gastroenterol. (1996) 23:44–6. 10.1097/00004836-199607000-00012 8835899

[41] Ludwig KA Milsom JW Church JM Fazio VW. Preliminary experience with laparoscopic intestinal surgery for Crohn's disease. Am J Surg. (1996) 171:52–5. 10.1016/S0002-9610(99)80073-7 8554151

[42] Shigeta K Okabayashi K Hasegawa H Tsuruta M Seishima R Kitagawa Y. Meta-analysis of laparoscopic surgery for recurrent Crohn's disease. Surg Today. (2016) 46:970–8. 10.1007/s00595-015-1271-7 26530516

[43] Young-Fadok TM HallLong K McConnell EJ Gomez Rey G Cabanela RL. Advantages of laparoscopic resection for ileocolic Crohn's disease. Improved outcomes and reduced costs. Surg Endosc. (2001) 15:450–4. 10.1007/s004640080078 11353959

[44] Milsom JW Hammerhofer KA Böhm B Marcello P Elson P Fazio VW. Prospective, randomized trial comparing laparoscopic vs. conventional surgery for refractory ileocolic Crohn's disease. Dis Colon Rectum. (2001) 44:1–8. 10.1007/BF02234810 11805557

[45] Benoist S Panis Y Beaufour A Bouhnik Y Matuchansky C Valleur P. Laparoscopic ileocecal resection in Crohn's disease: a case-matched comparison with open resection. Surg Endosc. (2003) 17:814–8. 10.1007/s00464-002-9103-4 12584603

[46] Duepree HJ Senagore AJ Delaney CP Brady KM Fazio VW. Advantages of laparoscopic resection for ileocecal Crohn's disease. Dis Colon Rectum. (2002) 45:605–10. 10.1007/s10350-004-6253-6 12004208

[47] Bergamaschi R Pessaux P Arnaud JP. Comparison of conventional and laparoscopic ileocolic resection for Crohn's disease. Dis Colon Rectum. (2003) 46:1129–33. 10.1007/s10350-004-7292-8 12907912

[48] Eshuis EJ Polle SW Slors JF Hommes DW Sprangers MA Gouma DJ. Long-term surgical recurrence, morbidity, quality of life, and body image of laparoscopic-assisted vs. open ileocolic resection for Crohnon disease: a comparative study. Dis Colon Rectum. (2008) 51:858–67. 10.1007/s10350-008-9195-6 18266036 PMC2440934

[49] Stocchi L Milsom JW Fazio VW. Long-term outcomes of laparoscopic versus open ileocolic resection for Crohn's disease: follow-up of a prospective randomized trial. Surgery. (2008) 144:622–7. 10.1016/j.surg.2008.06.016 18847647

[50] Lowney JK Dietz DW Birnbaum EH Kodner IJ Mutch MG Fleshman JW. Is there any difference in recurrence rates in laparoscopic ileocolic resection for Crohn's disease compared with conventional surgery? a long-term, follow-up study. Dis Colon Rectum. (2006) 49:58–63. 10.1007/s10350-005-0214-6 16328612

[51] Eshuis EJ Slors JF Stokkers PC Sprangers MA Ubbink DT Cuesta MA. Long-term outcomes following laparoscopically assisted versus open ileocolic resection for Crohn's disease. Br J Surg. (2010) 97:563–8. 10.1002/bjs.6918 20175126

[52] Dasari BV McKay D Gardiner K. Laparoscopic versus Open surgery for small bowel Crohn's disease. Cochrane Database Syst Rev. (2011) 19:Cd006956. 10.1002/14651858.CD006956.pub2 21249684 PMC13378878

[53] Tan JJ Tjandra JJ. Laparoscopic surgery for Crohn's disease: a meta-analysis. Dis Colon Rectum. (2007) 50:576–85. 10.1007/s10350-006-0855-0 17380366

[54] Patel SV Patel SV Ramagopalan SV Ott MC. Laparoscopic surgery for Crohn's disease: a meta-analysis of perioperative complications and long term outcomes compared with open surgery. BMC Surg. (2013) 13:14. 10.1186/1471-2482-13-14 23705825 PMC3733939

[55] Adamina M Bonovas S Raine T Spinelli A Warusavitarne J Armuzzi A. ECCO guidelines on therapeutics in Crohn's disease: surgical treatment. J Crohns Colitis. (2020) 14:155–68. 10.1093/ecco-jcc/jjz187 31742338

[56] Celentano V Pellino G Spinelli A Selvaggi F Celentano V Pellino G. Anastomosis configuration and technique following ileocaecal resection for Crohn's disease: a multicentre study. Updates Surg. (2021) 73:149–56. 10.1007/s13304-020-00918-z 33409848

[57] Riss S Bittermann C Zandl S Kristo I Stift A Papay P. Short-term complications of wide-lumen stapled anastomosis after ileocolic resection for Crohn's disease: who is at risk? Colorectal Dis. (2010) 12:e298–303. 10.1111/j.1463-1318.2009.02180.x 20041915

[58] Resegotti A Astegiano M Farina EC Ciccone G Avagnina G Giustetto A. Side-to-side stapled anastomosis strongly reduces anastomotic leak rates in Crohn's disease surgery. Dis Colon Rectum. (2005) 48:464–8. 10.1007/s10350-004-0786-6 15719193

[59] Yamamoto T Bain IM Mylonakis E Allan RN Keighley MR. Stapled functional end-to-end anastomosis versus sutured end-to-end anastomosis after ileocolonic resection in Crohn disease. Scand J Gastroenterol. (1999) 34:708–13. 10.1080/003655299750025921 10466883

[60] Hashemi M Novell JR Lewis AA. Side-to-side stapled anastomosis may delay recurrence in Crohn's disease. Dis Colon Rectum. (1998) 41:1293–6. 10.1007/BF02258231 9788394

[61] Kusunoki M Ikeuchi H Yanagi H Shoji Y Yamamura T. A comparison of stapled and hand-sewn anastomoses in Crohn's disease. Dig Surg. (1998) 15:679–82. 10.1159/000018677 9845636

[62] Ikeuchi H Kusunoki M Yamamura T. Long-term results of stapled and hand-sewn anastomoses in patients with Crohn's disease. Dig Surg. (2000) 17:493–6. 10.1159/000051946 11124554

[63] He X Chen Z Huang J Lian L Rouniyar S Wu X. Stapled side-to-side anastomosis might be better than handsewn end-to-end anastomosis in ileocolic resection for Crohn's disease: a meta-analysis. Dig Dis Sci. (2014) 59:1544–51. 10.1007/s10620-014-3039-0 24500450

[64] Feng JS Li JY Yang Z Chen XY Mo JJ Li SH. Stapled side-to-side anastomosis might be benefit in intestinal resection for Crohn's disease: a systematic review and network meta-analysis. Medicine (Baltimore). (2018) 97:e0315. 10.1097/MD.0000000000010315 29642162 PMC5908623

[65] Guo Z Li Y Zhu W Gong J Li N Li J. Comparing outcomes between side-to-side anastomosis and other anastomotic configurations after intestinal resection for patients with Crohn's disease: a meta-analysis. World J Surg. (2013) 37:893–901. 10.1007/s00268-013-1928-6 23354925

[66] McLeod RS Wolff BG Ross S Parkes R McKenzie M. Recurrence of Crohn's disease after ileocolic resection is not affected by anastomotic type: results of a multicenter, randomized, controlled trial. Dis Colon Rectum. (2009) 52:919–27. 10.1007/DCR.0b013e3181a4fa58 19502857

[67] Zurbuchen U Kroesen AJ Knebel P Betzler MH Becker H Bruch HP. Complications after end-to-end vs. side-to-side anastomosis in ileocecal Crohn's disease–early postoperative results from a randomized controlled multi-center trial (ISRCTN-45665492). Langenbecks Arch Surg. (2013) 398:467–74. 10.1007/s00423-012-0904-1 22290216

[68] Oresland T. Crohn's surgery: are there differences between the types of anastomosis? Inflamm Bowel Dis. (2008) 14(Suppl. 2):S273–4. 10.1002/ibd.20703 18816755

[69] Simillis C Purkayastha S Yamamoto T Strong SA Darzi AW Tekkis PP. meta-analysis comparing conventional end-to-end anastomosis vs. other anastomotic configurations after resection in Crohnon disease. Dis Colon Rectum. (2007) 50:1674–87. 10.1007/s10350-007-9011-8 17682822

[70] Gajendran M Bauer AJ Buchholz BM Watson AR Koutroubakis IE Hashash JG. Ileocecal anastomosis type significantly influences long-term functional status, quality of life, and healthcare utilization in postoperative Crohn's disease patients independent of inflammation recurrence. Am J Gastroenterol. (2018) 113:576–83. 10.1038/ajg.2018.13 29610509

[71] Tersigni R Alessandroni L Barreca M Piovanello P Prantera C. Does stapled functional end-to-end anastomosis affect recurrence of Crohn's disease after ileocolonic resection? Hepatogastroenterology. (2003) 50:1422–5. 14571753

[72] Smedh K Andersson M Johansson H Hagberg T. Preoperative management is more important than choice of sutured or stapled anastomosis in Crohn's disease. Eur J Surg. (2002) 168:154–7. 10.1080/110241502320127766 12182240

[73] Scarpa M Ruffolo C Bertin E Polese L Filosa T Prando D. Surgical predictors of recurrence of Crohn's disease after ileocolonic resection. Int J Colorectal Dis. (2007) 22:1061–9. 10.1007/s00384-007-0329-4 17534633

[74] Kono T Ashida T Ebisawa Y Chisato N Okamoto K Katsuno H. A new antimesenteric functional end-to-end handsewn anastomosis: surgical prevention of anastomotic recurrence in Crohn's disease. Dis Colon Rectum. (2011) 54:586–92. 10.1007/DCR.0b013e318208b90f 21471760

[75] Luglio G Kono T. Surgical Techniques and risk of postoperative recurrence in CD: a game changer? Inflamm Intest Dis. (2021). 10.1159/000515372. [Epub ahead of print]. PMC8820132 35224014

[76] Luglio G Tropeano FP Amendola A Rispo A Castiglione F Bucci L. Preventing recurrence in Crohn's disease: the Kono-S anastomosis with two-layer technique. Tech Coloproctol. (2020) 24:1213–4. 10.1007/s10151-020-02266-x 32564233

[77] Kono T Fichera A Maeda K Sakai Y Ohge H Krane M. Kono-S anastomosis for surgical prophylaxis of anastomotic recurrence in Crohn's disease: an international multicenter study. J Gastrointest Surg. (2016) 20:783–90. 10.1007/s11605-015-3061-3 26696531

[78] Luglio G Rispo A Castiglione F Imperatore N Giglio MC De Palma GD. Kono-type anastomosis in a patient with severe multi-recurrent Crohn's disease. Int J Colorectal Dis. (2016) 31:1565–6. 10.1007/s00384-016-2567-9 27026171

[79] Katsuno H Maeda K Hanai T Masumori K Koide Y Kono T. Novel antimesenteric functional end-to-end handsewn (Kono-S) anastomoses for Crohn's disease: a report of surgical procedure and short-term outcomes. Dig Surg. (2015) 32:39–44. 10.1159/000371857 25678306

[80] Fichera A Zoccali M Kono T. Antimesenteric functional end-to-end handsewn (Kono-S) anastomosis. J Gastrointest Surg. (2012) 16:1412–6. 10.1007/s11605-012-1905-7 22580840

[81] Seyfried S Post S Kienle P Galata CL. The Kono-S anastomosis in surgery for Crohn's disease: First results of a new functional end-to-end anastomotic technique after intestinal resection in patients with Crohn's disease in Germany. Chirurg. (2019) 90:131–6. 10.1007/s00104-018-0668-4 29931381

[82] Horisberger K Birrer DL Rickenbacher A Turina M. Experiences with the Kono-S anastomosis in Crohn's disease of the terminal ileum-a cohort study. Langenbecks Arch Surg. (2021) 406:1173–80. 10.1007/s00423-020-01998-6 33025079 PMC8208918

[83] Shimada N Ohge H Kono T Sugitani A Yano R Watadani Y. Surgical recurrence at anastomotic site after bowel resection in Crohn's disease: comparison of kono-s and end-to-end anastomosis. J Gastrointest Surg. (2019) 23:312–9. 10.1007/s11605-018-4012-6 30353491

[84] Luglio G Rispo A Imperatore N Giglio MC Amendola A Tropeano FP. Surgical prevention of anastomotic recurrence by excluding mesentery in crohn's disease: the supreme-CD study–a randomized clinical trial. Ann Surg. (2020) 272:210–7. 10.1097/SLA.0000000000003821 32675483

[85] Peltrini R Greco PA Manfreda A Luglio G Bucci L. Kono-S anastomosis after intestinal resection for Crohn's disease. Updates Surg. (2020) 72:335–40. 10.1007/s13304-019-00700-w 31897890

[86] Alshantti A Hind D Hancock L Brown SR. The role of Kono-S anastomosis and mesenteric resection in reducing recurrence after surgery for Crohn's disease: a systematic review. Colorectal Dis. (2021) 23:7–17. 10.1111/codi.15136 32418300

[87] Ng CH Chin YH Lin SY Koh JWH Lieske B Koh FH. Kono-S anastomosis for Crohn's disease: a systemic review, meta-analysis, and meta-regression. Surg Today. (2021) 51:493–501. 10.1007/s00595-020-02130-3 32894346

[88] Smedh K Olaison G Sjödahl R. Ileocolic nipple valve anastomosis for preventing recurrence of surgically treated Crohn's disease. Long-term follow-up of six patients. Dis Colon Rectum. (1990) 33:987–90. 10.1007/BF02139113 2226091

[89] Sjödahl R. Can ileocolic nipple valve anastomosis prevent recurrence of Crohn's disease? Ann Med. (1992) 24:423–4. 10.3109/07853899209166989 1485933

[90] Gustafson C Sjödahl R Tagesson C. Phospholipase activation and arachidonic acid release in intestinal epithelial cells from patients with Crohn's disease. Scand J Gastroenterol. (1990) 25:1151–60. 10.3109/00365529008998548 2274737

[91] Kald B Olaison G Sjödahl R Tagesson C. Novel aspect of Crohn's disease: increased content of platelet-activating factor in ileal and colonic mucosa. Digestion. (1990) 46:199–204. 10.1159/000200346 2282995

[92] Bakkevold KE. Nipple valve anastomosis for preventing recurrence of Crohn disease in the neoterminal ileum after ileocolic resection. A prospective pilot study. Scand J Gastroenterol. (2000) 35:293–9. 10.1080/003655200750024173 10766324

[93] Bakkevold KE. Construction of an ileocolic neosphincter–Nipple valve anastomosis for prevention of postoperative recurrence of Crohn's disease in the neoterminal ileum after ileocecal or ileocolic resection. A long-term follow-up study. J Crohns Colitis. (2009) 3:183–8. 10.1016/j.crohns.2009.04.002 21172268

[94] Coffey JC O'Leary DP. The mesentery: structure, function, and role in disease. Lancet Gastroenterol Hepatol. (2016) 1:238–47. 10.1016/S2468-1253(16)30026-7 28404096

[95] Coffey JC O'Leary D P. Defining the mesentery as an organ and what this means for understanding its roles in digestive disorders. Expert Rev Gastroenterol Hepatol. (2017) 11:703–5. 10.1080/17474124.2017.1329010 28482706

[96] Coffey JC O'Leary DP Kiernan MG Faul P. The mesentery in Crohn's disease: friend or foe? Curr Opin Gastroenterol. (2016) 32:267–73. 10.1097/MOG.0000000000000280 27115218

[97] Rivera ED Coffey JC Walsh D Ehrenpreis ED. The Mesentery, Systemic Inflammation, and Crohn's Disease. Inflamm Bowel Dis. (2019) 25:226–34. 10.1093/ibd/izy201 29920595

[98] Wickramasinghe D Warusavitarne J. The role of the mesentery in reducing recurrence after surgery in Crohn's disease. Updates Surg. (2019) 71:11–2. 10.1007/s13304-019-00623-6 30712254

[99] Peltrini R Bucci L. “Mesentery-based surgery” to prevent surgical recurrence in Crohn's disease: from basics to surgical practice. Int J Colorectal Dis. (2019) 34:353–4. 10.1007/s00384-018-3197-1 30417271

[100] Li Y Zhu W Zuo L Shen B. The role of the mesentery in Crohn's disease: the contributions of nerves, vessels, lymphatics, and fat to the pathogenesis and disease course. Inflamm Bowel Dis. (2016) 22:1483–95. 10.1097/MIB.0000000000000791 27167572

[101] de Groof EJ van der Meer JHM Tanis PJ de Bruyn JR van Ruler O D'Haens G. Persistent mesorectal inflammatory activity is associated with complications after proctectomy in Crohn's disease. J Crohns Colitis. (2019) 13:285–93. 10.1093/ecco-jcc/jjy131 30203027

[102] Coffey CJ Kiernan MG Sahebally SM Jarrar A Burke JP Kiely PA. Inclusion of the mesentery in ileocolic resection for crohn's disease is associated with reduced surgical recurrence. J Crohns Colitis. (2018) 12:1139–50. 10.1093/ecco-jcc/jjx187 29309546 PMC6225977

[103] Buskens CJ Bemelman WA. Inclusion of the mesentery in ileocolic resection for crohn's disease is associated with reduced surgical recurrence: editorial by coffey et al. J Crohns Colitis. (2018) 12:1137–8. 10.1093/ecco-jcc/jjy115 30137343

[104] Zhu Y Qian W Huang L Xu Y Guo Z Cao L. Role of extended mesenteric excision in postoperative recurrence of crohn's colitis: a single-center study. Clin Transl Gastroenterol. (2021) 12:e00407. 10.14309/ctg.0000000000000407 34597277 PMC8483874

[105] Valibouze C Desreumaux P Zerbib P. Post-surgical recurrence of Crohn's disease: situational analysis and future prospects. J Visc Surg. (2021) 158:401–10. 10.1016/j.jviscsurg.2021.03.012 33858790

[106] Martins R Carmona C George B Epstein J. Management of Crohn's disease: summary of updated NICE guidance. Bmj. (2019) 367:l5940. 10.1136/bmj.l5940 31676715

[107] Preiß JC Bokemeyer B Buhr HJ Dignaß A Häuser W Hartmann F. [Updated German clinical practice guideline on “Diagnosis and treatment of Crohn's disease” 2014]. Z Gastroenterol. (2014) 52:1431–84. 10.1055/s-0034-1385199 25474283

[108] Aratari A Papi C Leandro G Viscido A Capurso L Caprilli R. Early versus late surgery for ileo-caecal Crohn's disease. Aliment Pharmacol Ther. (2007) 26:1303–12. 10.1111/j.1365-2036.2007.03515.x 17848181

[109] Kelm M Germer CT Schlegel N Flemming S. The revival of surgery in Crohn's disease-early intestinal resection as a reasonable alternative in localized ileitis. Biomedicines. (2021) 9:1317. 10.3390/biomedicines9101317 34680434 PMC8533348

[110] de Groof EJ Stevens TW Eshuis EJ Gardenbroek TJ Bosmans JE van Dongen JM. Cost-effectiveness of laparoscopic ileocaecal resection versus infliximab treatment of terminal ileitis in Crohn's disease: the LIR!C Trial. Gut. (2019) 68:1774–80. 10.1136/gutjnl-2018-317539 31233395

[111] Stevens TW Haasnoot ML D'Haens GR Buskens CJ de Groof EJ Eshuis EJ. Laparoscopic ileocaecal resection versus infliximab for terminal ileitis in Crohn's disease: retrospective long-term follow-up of the LIR!C trial. Lancet Gastroenterol Hepatol. (2020) 5:900–7. 10.1016/S2468-1253(20)30117-5 32619413

[112] Bucala R Spiegel LA Chesney J Hogan M Cerami A. Circulating fibrocytes define a new leukocyte subpopulation that mediates tissue repair. Mol Med. (1994) 1:71–81. 10.1007/BF03403533 8790603 PMC2229929

[113] Eder P Adler M Dobrowolska A Kamhieh-Milz J Witowski J. The role of adipose tissue in the pathogenesis and therapeutic outcomes of inflammatory bowel disease. Cells. (2019) 8:628. 10.3390/cells8060628 31234447 PMC6627060

[114] Hollis RH Smith N Sapci I Click B Regueiro M Hull T. Small bowel Crohn's disease recurrence is common after total proctocolectomy for Crohn's colitis. Dis Colon Rectum. (2021). 10.1097/DCR.0000000000002328. [Epub ahead of print]. 34759246

[115] Daoud ND Hashash JG Picco MF Farraye FA. Fecal calprotectin from ileostomy output is sensitive and specific for the prediction of small bowel inflammation in patients with Crohn's disease. J Crohns Colitis. (2021). 10.1093/ecco-jcc/jjab182. [Epub ahead of print]. 34633435

